# Contamination with recombinant IFN accounts for the unexpected stimulatory properties of commonly used IFN-blocking antibodies

**DOI:** 10.1002/eji.200940145

**Published:** 2010-11-16

**Authors:** Herwig P Moll, Harald Freudenthaler, Anna Zommer, Elisabeth Buchberger, Xiao-Hong Lin, Sara Crisafulli, Yognandan Pandya, Sidney Pestka, Thomas Lavoie, Christine Brostjan

**Affiliations:** 1 Department of Surgery, Medical University of Vienna, General HospitalVienna, Austria; 2 PBL InterferonSourcePiscataway, NJ, USA

**Keywords:** Antibodies, Cellular activation, Cytokines

The cellular response to IFN is an essential part of immune reactions and has been subject to investigations for over 50 years 1. The analyses on IFN function frequently involve the use of neutralizing antibodies to block responses and to document the dependence on IFN signals. In this context, we have previously described an unusual “IFN-like” response initiated by blocking antibodies to type I IFN in primary human endothelial cells (EC) or mononuclear blood cells. In the absence of exogenously added recombinant IFN (rIFN), the exposure of EC to increasing concentrations of IFN-blocking mAb resulted in the dose-dependent induction of IFN response genes at the mRNA and protein level 2. The effect was observed for four different mAb directed against human IFN-α or -β and was dependent on the type I IFN receptor. We concluded that an intrinsic feature of the IFN-blocking antibodies was responsible for the observed “IFN-like” activation of EC; a model was proposed of antibody binding to surface Fc-receptors with sequestration of autocrine IFN and subsequent release to nearby IFN receptors, which would result in the observed “IFN-like” signal.

We have now obtained evidence that refutes this hypothesis showing that the “IFN-like” activity associated with IFN-blocking mAb is indeed a discrete component that can be separated from the antibody moiety by sequential cycles of antibody immunoprecipitation (Supporting Information Fig. 1 and Supporting Information Methodology). Furthermore, when the standard two-step procedure for antibody purification as performed by the manufacturer (based on ammonium sulfate precipitation and ion exchange chromatography) was extended by a third step of hydrophobic interaction chromatography, the “IFN-like” activity was lost and the neutralizing capacity of the respective antibodies prevailed (Fig. 1A–C).

**Figure 1:**
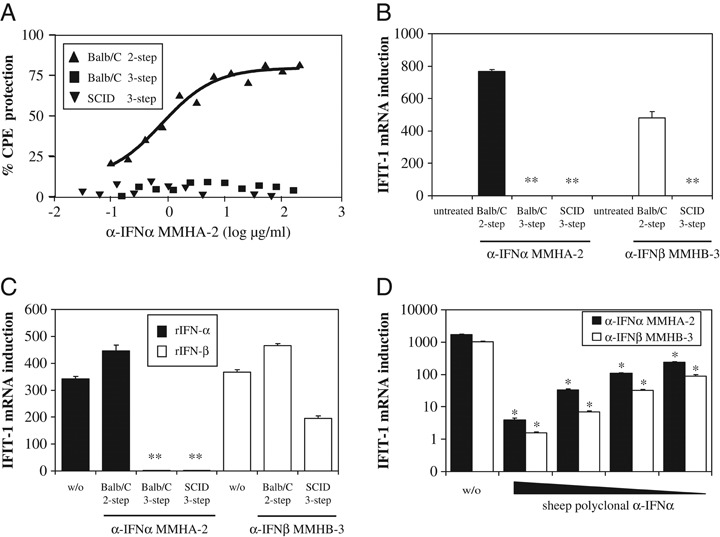
The “IFN-like” activity in antibody preparations can be eliminated by additional antibody purification (three-step process) and is inhibited by polyclonal anti-IFN-α antiserum. The neutralizing anti-IFN-α mAb MMHA-2 and the anti-IFN-β mAb MMHB-3 were isolated from BALB/c or SCID mouse ascites by a standard two-step procedure or an enhanced three-step protocol that included hydrophobic interaction chromatography. (A) Antibody preparations were titrated in a standard A549/EMCV cytopathic effect inhibition assay using a calibrated standard of human IFN-α_2a_ as a reference (see Supporting Information *Materials and methods* and Table 1). The “IFN-like” antiviral activity of antibody preparations was determined as the percentage of cells protected from the virus-mediated cytopathic effect in relation to antibody concentration. Experiments were performed eight times for MMHA-2 (one representative set of data shown) and three times for MMHB-3 (data not shown). (B) Antibody preparations were further applied to stimulate EC for 4 h (at 6 μg/mL corresponding to 10 pg mAb *per* cell). Stimulation was assessed by the level of mRNA expression of the IFN-responsive gene IFIT-1 as measured by quantitative RT-PCR and is represented in fold induction relative to untreated control cells. (C) EC were also stimulated with rIFN-α_2a_ or rIFN-β (100 pg/mL) for 4 h in the absence or presence of the neutralizing antibody preparations (6 μg/mL) and the level of IFIT-1 mRNA was determined. (B and C) The experiments were performed three to four times in triplicate; data shown are the mean +SD of triplicates for one representative experiment; statistically significant differences between two-step and three-step antibody preparations were based on all experiments performed. (D) EC were stimulated with MMHA-2 or MMHB-3 (6 μg/mL) in the presence of decreasing concentrations of neutralizing sheep polyclonal anti-human IFN-α antiserum (dilutions of 1:800, 1600, 3200, 6400). Data from three independent experiments were evaluated for differences in IFIT-1 expression, comparing MMHA-2 (and MMHB-3) in the presence and absence of sheep polyclonal IFN-α antiserum. * *p*≤0.05, ^**^p≤0.01 (Wilcoxon test).

Having established that the “IFN-like” activity was attributable to a discrete contaminant of the applied anti-IFN antibody preparations, the possible contamination with microbial products was first examined. Since the majority of pathogen-associated signals leading to the IFN pathway are mediated by the TLR family 3, 4 we screened for hallmarks of TLR activity. However, we did not observe the induction of the transcription factor NF-κB or the pro-inflammatory activation of EC, strongly arguing against TLR involvement (Supporting Information Fig. 2).

We then obtained an indication towards contamination with type I IFN from competition studies showing that the contaminant in mAb preparations was neutralized by rabbit (data not shown) or sheep polyclonal anti-human IFN-α antiserum (Fig. 1D). Polyclonal anti-IFN-β antiserum or control antiserum obtained prior to immunization did not affect the “IFN-like” activity (data not shown).

The co-purification (and cross-reactivity) of mouse IFN upon mAb isolation from mouse ascites was a potential source of contamination, which was addressed by cytopathic effect inhibition assays on mouse *versus* human target cells. There was a significantly higher impact on the human target cells, thus arguing for the presence of human rather than mouse IFN-α (Supporting Information Fig. 3A).

However, the question remained as to why the contaminating human IFN-α was not neutralized by the investigated anti-IFN-α-blocking mAb (*e.g*. MMHA-2). When comparing the neutralizing capacity towards various rIFN-α subtypes, the three-step purified mAb failed to inhibit individual family members (IFN-α subtypes 8, 14, and 16) while the sheep polyclonal antiserum potently repressed all IFN-α subtypes (Supporting Information Fig. 3C). This finding supported the notion that a distinct human IFN-α subtype not neutralized by the respective monoclonal was present in the antibody preparation. In accordance, we found that the purified mAb could not block the “IFN-like” activity present in the contaminated mAb preparation (Supporting Information Fig. 3B).

Of note, rIFN-α_8_ and rIFN-α_14_ had been produced by PBL prior to the preparation of the contaminated antibody MMHA-2. By applying two anti-human IFN-α ELISA tests (not mouse cross-reactive) with distinct sensitivity towards rIFN-α_8_ and rIFN-α_14_ evidence was gained for a predominant antibody contamination by human rIFN-α_14_ (Supporting Information Table 1). However, a combination of contaminating IFN cannot be excluded.

Thus, the source of contamination could be traced to the sequential production of rIFN and anti-IFN-blocking antibodies with common equipment. Despite a time window of several months between productions, despite the regular two-step purification procedure, and despite standard equipment cleansing, the contamination of antibody preparations with functional type I IFN was substantial.

The importance of our observation was further demonstrated by the frequent occurrence of detectable IFN activity in a considerable number of tested antibodies (Table 1). Apart from various mouse monoclonals against human IFN-α and IFN-β (MMHA-2, MMHA-3, MMHA-9, MMHA-13, MMHB-3, MMHB-12), rat anti-mouse antibodies directed against IFN-α (RMMA-1) or IFN-γ (RMMG-1) also presented with significant amounts of human rIFN. While most of these monoclonals originated from PBL and were supplied by PBL or associated distributors in the contaminated form, further examples for contaminated antibodies were found for an alternative supplier. Two anti-pig IFN mAb (K9, F17) similarly showed contamination with rIFN-α (Table 1). Based on the diverse specificity of contaminated antibodies we propose that unspecific co-purification rather than specific antibody binding accounts for the presence of contaminants. While most of the affected antibodies showed IFN-α contamination, the subtype present may vary.

**Table 1:** Level of detectable contamination with rIFN-α in various antibody preparationsa)

Monoclonal antibody	Antiviral activity (U/mL)	IFN-α detected by ELISA (ng/mL)	Specific activity (U/mg)	Total protein in mAb preparation (mg/mL)	Contamination with rIFN-α (%)
α-hIFNb)-α MMHA-2	125 000	36.7	3.4E+09	7.5	4.9E−04
α-hIFN-α MMHA-9	92 000	50.0	1.8E+09	4.5	1.1E−03
α-hIFN-α MMHA-13	750	<0.1	> 6E+09	4.2	<3.0E−06
α-hIFN-β MMHB-3	1282	4.0	3.2E+08	3.6	1.1E−04
α-pigIFN-α K9	1200	3.0	4.0E+08	4.4	6.8E−05
α-pigIFN-α F17	140	0.1	1.1E+09	4.4	<3.0E−06

a) The antibody preparations were tested in the human A549/EMCV cytopathic effect inhibition assay and antiviral activity was expressed in equivalents of recombinant human IFN-α_2a_ (U/mL). Concentrations of rIFN-α (ng/mL) were measured by ELISA ♯41105 (PBL InterferonSource) and applied to calculate the specific IFN activity in U/mg. To determine the percentage of contamination, the detected IFN-α level was set in relation to the total amount of protein in antibody preparations.

b) hIFN: human IFN.

The range of detectable IFN activity varied considerably (by a factor of 1000). The highest levels of anti-viral activity as recorded for the anti-IFN-α mAb clone MMHA-2 equalled a concentration of 800 U/mL of human rIFN-α when applying the antibody at a dilution of 50 μg/mL (common for *in vitro* experiments). For example, stimulation of target cells with 1000 U/mL of biological or rIFN-α_2a_ in the presence of 50 μg/mL of contaminated blocking mAb MMHA-2 would be expected to result in the complete neutralization of the α_2a_ subtype, while exposing the cells to 800 U/mL of non-neutralized rIFN-α_14_. The net inhibitory effect on the target cells would be minor leading to the false interpretation of results, especially for an experimental setup where the involvement and concentration of type I IFN is the unknown parameter under investigation. Thus, the information given in this report may be of help in interpreting previously conducted experiments with the listed antibodies.

With respect to PBL products, all mAb preparations have been carefully evaluated, and contaminated antibodies were found to date back to the last 2–8 years. More stringent purification and equipment cleaning procedures as well as routine testing for contaminating activity have been put in place at PBL in part due to these experiments. With respect to K9, F17, the company producing these antibodies was informed and has, in the meantime, provided the respective clones to PBL for antibody production. Hence, all products identified in this report to have previously been affected by contamination are now being supplied to the research community in a purified form; however, it is easy to envision that reagent providers who prepare multiple cytokines and mAb could face similar issues as those noted here.

## Abbreviations

EC: endothelial cell
rIFN: recombinant IFN

## Supplementary material

Detailed facts of importance to specialist readers are published as ”Supporting Information”. Such documents are peer-reviewed, but not copy-edited or typeset. They are made available as submitted by the authors.
