# Supplementary material for: Contamination with recombinant IFN accounts for the unexpected stimulatory properties of commonly used IFN-blocking antibodies
Source: Eur J Immunol. 2010 Nov 16;41(1):252–4. doi: 10.1002/eji.200940145 (PMC3437480; doi:10.1002/eji.200940145)
Supplement: Supplementary file 1 [file eji0041-0252-SD1.pdf]

European Journal of Immunology

Supporting Information

for

DOI 10.1002/eji.200940145

Contamination with recombinant IFN accounts for the unexpected stimulatory properties of commonly used IFN-blocking antibodies

Herwig P. Moll, Harald Freudenthaler, Anna Zommer, Elisabeth Buchberger, Xiao-Hong Lin, Sara Crisafulli, Yognandan Pandya, Sidney Pestka, Thomas Lavoie and Christine Brostjan

© 2010 WILEY-VCH Verlag GmbH & Co. KGaA, Weinheim

Supplementary materials and methods

Endothelial cell isolation, characterization and culture

Primary EC were isolated from human foreskin samples by dispase digest, purified via α-CD31 antibody coupled Dynabeads (Invitrogen Corp., Carlsbad, CA, USA) and cultured in fibronectin-containing EGM2-MV growth medium (Cambrex Corp., East Rutherford, NJ, USA) without VEGF supplementation. Purified EC cultures showed ≥ 98% purity and viability as characterized by immunostaining with fluorescence-labeled antibodies (AbD Serotec, Oxford, UK) and flow cytometry for EC markers CD31, CD34, CD54 and CD62E (E-selectin) following stimulation with 100 ng/ml of TNFα or 1 µg/ml of LPS for 4 h. To keep the passage number low, experiments were generally repeated with different EC isolates, i.e., represent biological replicates with variable inducibility. Representative results of 2-3 comparable experiments are shown (as further specified in the figure legends).

Treatment with neutralizing antibodies and rIFN

Two days prior to stimulation, EC were seeded in growth medium at 1.25×10^5 cells per 15 mm dish (24 well plate) to yield a confluent cell layer within 24 h. Culture medium was then exchanged for EGM2-MV containing 5% FBS but no additional growth factor supplements and cells were allowed to adopt a quiescent state over the next 24 h. Antibodies and recombinant IFN were added to 200 µl conditioned medium on day 3 at the indicated concentrations and cells were generally stimulated for 4 h.

Recombinant interferons, rabbit or sheep polyclonal human IFN-α antiserum and blocking mAb targeting either human IFN-α (clones MMHA-2, MMHA-9, and MMHA-13) or IFN-β (clone MMHB-3) were all from PBL Biomedical Laboratories. ELISA assays for detection of human IFN-α were also manufactured by PBL and carried out essentially as specified. For mouse IgG_1 isotype control the MOPC-21 clone was applied (Sigma-Aldrich Corp., St. Louis, MO, USA). Further reagents obtained from Sigma-Aldrich include LPS and polymyxin B; TNFα was kindly provided by H. R. Alexander (NCI, NIH, Bethesda, MD, USA).

Immunoprecipitation and immunoblotting

Anti-IFN-α mAb clone MMHA-2 (6 µg) was diluted in 1 ml of EGM2-MV medium (without supplementation of growth factors). Three steps of immunoprecipitation were performed by addition of 20 µl protein A/G PLUS agarose (Santa Cruz Biotechnology, Inc., Santa Cruz, CA, USA), incubation at $4^{o}C$ for 1 h and centrifugation for 5 min at 2000 x g and $4^{o}C$. At each step, 200 µl of supernatant as well as the IP pellet were set aside for subsequent analysis. The remaining supernatant was again supplied with 20 µl protein A/G PLUS agarose for the next IP cycle. For EC stimulation, 180 µl of IP supernatant were directly applied to endothelial cultures for 4 hours, prior to RNA isolation and quantitative RT-PCR analysis. For Western blot analysis, 10 µl of IP supernatant or original antibody solution (equaling 60 ng) were separated on PAGE mini gels and subjected to semi-dry blotting. IP pellets were treated with 20 µl of SDS/PAGE

loading buffer (for 5 min at 95°C) to retrieve bound proteins; 10 µl thereof were also analyzed by Western blotting with ImmunoPure peroxidase-conjugated goat anti-mouse IgG (H&L chain) antiserum (Pierce Biotechnology, Rockford, IL, USA).

Real-time RT-PCR

Endothelial RNA was isolated with E.Z.N.A. Total RNA Kit (Omega Bio-tek, Inc., Norcross, GA, USA), 500 ng of RNA were reverse transcribed with oligo(dT) primers using the Superscript III First Strand Synthesis System (Invitrogen) and the generated cDNA was diluted 1:25 prior to PCR analysis. Real-time PCR was performed with the qPCR MasterMix Plus Low ROX (Eurogentec, Seraing, Belgium) and the following probe (200 nM) and primer (450 nM) sets for IFIT-1 (forward primer 5'-GAT CTC AGA GGA GCC TGG CTA A-3', reverse primer 5'-TGA TCA TCA CCA TTT GTA CTC ATG G-3', YakimaYellow/BHQ-1 labeled probe 5'-CAA AAC CCT GCA GAA CGG CTG CC-3'), ISG-15 (forward primer 5'-GAG AGG CAG CGA ACT CAT CT-3', reverse primer 5'-AGG GAC ACC TGG AAT TCG TT-3', 6-FAM/BHQ-1 labeled probe 5'-TGC CAG TAC AGG AGC TTG TG-3'), housekeeping genes β_2-microglobulin (forward primer 5'-CGC TCC GTG GCC TTA GC-3', reverse primer 5'-AAT CTT TGG AGT ACG CTG GAT AGC-3', YakimaYellow/BHQ-1 labeled probe 5'-TGC TCG CGC TAC TCT CTC TTT CTG GC-3') and β-actin (forward primer 5'-CCT GGC ACC CAG CAC AAT-3', reverse primer 5'-GCC GAT CCA CAC GGA GTA CT-3', 6-FAM/BHQ-1

labeled probe 5’-ATC AAG ATC ATT GCT CCT CCT GAG CGC-3’). Simultaneous detection (multiplexing) of IFIT-1 and ISG15 as well as of β_2-microglobulin and β-actin PCR products was performed. Each sample was assayed in triplicate with the 7500 Fast PCR Detection System (Applied Biosystems, Foster City, CA) for 45 cycles of 3 sec at 95ºC followed by 30 sec at 60ºC. mRNA levels for IFIT-1 and ISG15 were deduced from the on-plate dilution series of a standard cDNA and were normalized to the respective housekeeping gene values. Real-time PCR data are given as mean and standard deviation of triplicate samples. The value obtained for the untreated control sample was set to 1 and changes in mRNA expression upon stimulation are given in relation to the untreated control. While data are generally shown for IFIT-1 transcripts, all experiments have been evaluated for IFIT-1 and ISG15 regulation yielding comparable results.

NF-κB Reporter Gene Assay

Electroporation of $2x10^6$ EC at 200 V, 960 µF was performed with a total of 30 µg DNA. A combination of 29 µg reporter plasmid with 3 NF-κB binding sites directing expression of the luciferase reporter gene [1] and 1 µg of the constitutive β-galactosidase expression plasmid pCMVβ for normalization (Clontech Laboratories, Inc., Mountain View, CA, USA) was applied. Cells were then seeded in 30 mm wells at $1x10^6$ to yield a confluent cell layer within 24 hours. Stimulation with anti-IFN-α mAb MMHA-2 (6 µg/ml), TNFα (100 ng/ml) or rIFN-α_{2a} (100 pg/ml) was carried out for 4 and 8 hours. Cells were then harvested

in 50 µl phosphate buffer and exposed to 3 cycles of freeze-thaw lysis. Samples (supernatant à 10 µl) were assayed in triplicate for luciferase as well as β-galactosidase activity applying the Tropix Dual-Light System according to manufacturer's instructions (Applied Biosystems) for chemiluminescent detection with a Wallac Victor^3 multilabel counter (Perkin Elmer Life Sciences, Waltham, MA, USA). Luciferase activity as measured in relative light units was normalized to the corresponding β-galactosidase value and expressed as fold induction compared to the non-stimulated control.

Cytopathic effect inhibition assay

Antiviral assays were conducted on A549 human lung carcinoma (ATCC CCL-185) and L929 murine fibroblast (ATCC CCL-1) cells. For the A549 assay, 10000 cells in 0.1 ml of DMEM with 10% FBS were plated in each well of a 96 well tissue culture plate and incubated at 37°C with 5% CO_2 for 4 hours. Serial dilutions of the test samples (interferon or antibody preparations) were prepared in media and 0.1 ml was transferred to the plate containing the cells. Additional wells containing no interferon were included. After incubation for 18 hours a dilution of murine encephalomyocarditis virus (EMCV, ATCC VR-129B) which had been empirically determined to yield 100% cytopathic effect in 40 hours, was added in 0.05 ml of media. Wells which received virus but no IFN served as the viral killing control, wells receiving 0.05 ml of media but no virus or IFN served as the cell control. The assay was allowed to develop for 40-48 hours in the incubator; the remaining cells were stained with crystal violet. After air drying the

plates were read visually and then the crystal violet was solubilized with 0.1 ml of 70% methanol; the absorbance at 570 nm was determined in a Molecular Devices (Sunnyvale, CA, USA) VMax microplate reader. CPE protection was determined relative to the cell control (100%) and virus control (0%) and the EC50 was determined in U/ml relative to a laboratory standard of human IFN-α_{2a} calibrated to the international standard Gxa01-901-535 for human IFN-α (BEI Resources, Manassas, VA, USA).

The L929 assay was performed in a similar manner with the following exceptions. The media used was MEM with 10% FBS, 30000 cells were plated in 0.1 ml, the virus was added and the assay allowed to resolve for 24 hours and the standard used to calibrate the samples was murine IFN-alpha A calibrated versus the international standard for murine IFN-α (BEI Resources Ga02-901-511).

Antibody purification

All antibodies were purified from mouse ascites fluid. Two procedures were used. A 2-step procedure consisting of ammonium sulphate precipitation and ion exchange chromatography was applied. The three step procedure used for later preparations involved the first two steps followed by hydrophobic charge induction chromatography. Frozen ascites fluid was thawed and filtered through 2 layers of cheese cloth. The ascites fluid was diluted with 2 volumes of dPBS and ammonium sulfate precipitation was performed at 1.85 M final concentration, incubation on ice for 1 hour and centrifugation. The precipitate was resuspended in dPBS and the precipitation step was repeated. The final pellet was resuspended in 50 mM Tris pH 8.3, 40 mM NaCl and dialyzed extensively versus this buffer.

The dialysate was loaded onto a 100 ml packed volume of Diethylaminoethyl-ToyoPearl (Tosoh Bioscience, Tokyo, Japan), washed with 5 column volumes of buffer and eluted with a 40 mM to 500 mM gradient of NaCl over 20 column volumes. Samples containing monoclonal antibody were identified by NuPAGE Bis-Tris electrophoresis, and were then dialyzed versus storage buffer and frozen.

The third step used on later batches was MEP-HyperCel™ (Pall Corp., East Hills, NY, USA). Eluted mAb from the diethylaminoethyl column was adjusted to 0.5 M NaCl, was loaded onto a 25 ml MEP-HyperCel column and washed with 5 column volumes of the same buffer. Elution was performed step-wise with 5 column volumes each of dPBS, 50 mM citrate-phosphate buffer pH 6.0, 5.5, 5.0, 4.5, 4.0 and 2.5. Fractions containing mAb were determined by gel electrophoresis followed by direct binding ELISA. Briefly, Costar Hi Bind Strip wells (Corning Incorporated, Corning, NY, USA) were coated with 1 μg/ml IFN and blocked with 1% BSA/PBS. Serial dilutions of the mAb in 0.1% BSA/PBS/0.05% Tween were applied. The plates were washed twice with PBS/0.05% Tween and bound antibody was detected by incubation of a 1:10000 dilution of donkey anti-mouse IgG-HRP conjugate followed by 3 washes and detection with 3,3´,5,5´-tetramethylbenzidine substrate. Color evolution was stopped by acidification and absorbance read at 450 nm in a VMax plate reader (Molecular Devices). Active fractions were pooled and frozen for long term storage.

Reference

[1] **Brostjan, C.** ***et al.*****,** *J. Immunol.* 1997. **158**: 3836-3844.

Supplementary figure legends

Supplementary figure 1. The “IFN-like” activity of neutralizing IFN-antibody preparations is separable from the antibody moiety. The anti-IFN-α mAb clone MMHA-2 (6 µg in 1 ml EC medium) was subjected to three rounds of immunoprecipitation using protein A/G PLUS agarose. Aliquots of the supernatant as well as the bead pellet were retrieved after each step of one experiment. (A) Western blot analysis of the supernatants and precipitated (IP) proteins was performed with goat anti-mouse IgG antiserum to detect the heavy (55 kDa) and light (25 kDa) chains of the monoclonal antibody. For comparison, preparations of MMHA-2 (6 µg/ml and 1 µg/ml) were loaded. (B) The IP supernatant was also applied to EC cultures in comparison to the original MMHA-2 antibody preparation (at 6 or 1 µg/ml). Endothelial RNA was isolated 4 h thereafter for quantitative RT-PCR analysis of IFIT-1 expression reflecting the “IFN-like” endothelial cell activation. Induction of IFIT-1 mRNA is calculated in relation to untreated control cells.

Supplementary figure 2. The contaminant in neutralizing IFN-antibody preparations has no pro-inflammatory activity indicative of TLR involvement. Endothelial cell activation by the TLR4 agonist LPS was compared to EC stimulation with anti-IFN-α antibody or recombinant interferon. EC were left untreated or were exposed to the LPS antagonist polymyxin B (PMB) at 50 µg/ml for 30 min prior to stimulation with LPS (1 µg/ml), with anti-IFN-α mAb clone MMHA-2 (6 µg/ml) or rIFN-α_{2a} (100 pg/ml) for 4 h. All experiments were

performed twice. (A) Cells were harvested for CD62E surface staining and flow cytometric analysis to assess induction of the pro-inflammatory adhesion molecule E-selectin (CD62E). (B) Additionally, EC RNA was analyzed by quantitative RT-PCR for the expression of interferon-responsive genes IFIT-1 and ISG15 in relation to untreated control cells. Data depict ISG15 transcript levels, as IFIT-1 mRNA expression was not regulated by LPS treatment. (C) The key transcription factor NF-κB generally activated by TLR pathways was investigated by reporter assay. EC were transfected with an NF-κB reporter plasmid and subsequently stimulated with MMHA-2 (6 µg/ml), TNFα (100 ng/ml) or rIFN-α_{2a} (100 pg/ml) for 4 and 8 hours. Reporter gene expression is given in fold induction compared to non-stimulated control cells. All data presented are mean + SD of triplicate samples of one representative experiment.

Supplementary figure 3. The "IFN-like" activity in neutralizing antibody preparations is likely to be due to contamination with distinct human IFN-α subtypes. (A) To evaluate whether the contamination was of human or mouse origin, two antibody preparations (anti-IFN-α mAb MMHA-2 and MMHA-9) were tested in a standard human (A549/EMCV) versus mouse cell (L929/EMCV) cytopathic effect inhibition assay. The anti-viral activity of antibody preparations was determined in percentage of cells protected from the virus-mediated cytopathic effect in relation to antibody concentration. The equivalent to protection by recombinant (human or mouse) IFN-α was calculated in U/ml for the original antibody preparations. The data depict the MMHA-2 assay; values obtained for MMHA-9 were 9.20E+04 U/ml and <3.6E+01 U/ml in human and

mouse assay, respectively. (B) In direct competition assays (*n* = 2) neutralization of the contaminant in 2-step purified anti-IFN-α mAb MMHA-2 (3 μg/ml) was tested by addition of 3-step purified MMHA-2 and/or anti-IFN-β mAb clone MMHB-3 (at 1, 3, and 6 μg/ml) and was monitored by IFIT-1 mRNA expression after 4 h of EC treatment. (C) In two sets of experiments comparing the neutralizing capacity and specificity of the monoclonal versus polyclonal antibodies, EC were stimulated for 4 h with rIFN-α (100 pg/ml) of all known subtypes, in the absence or presence of 3-step purified anti-IFN-α mAb MMHA-2 (3 μg/ml) or sheep polyclonal anti-human IFN-α antiserum (at 1:800 dilution). Data in B and C are mean + SD of triplicate samples for one representative experiment.

Supplementary figure 1

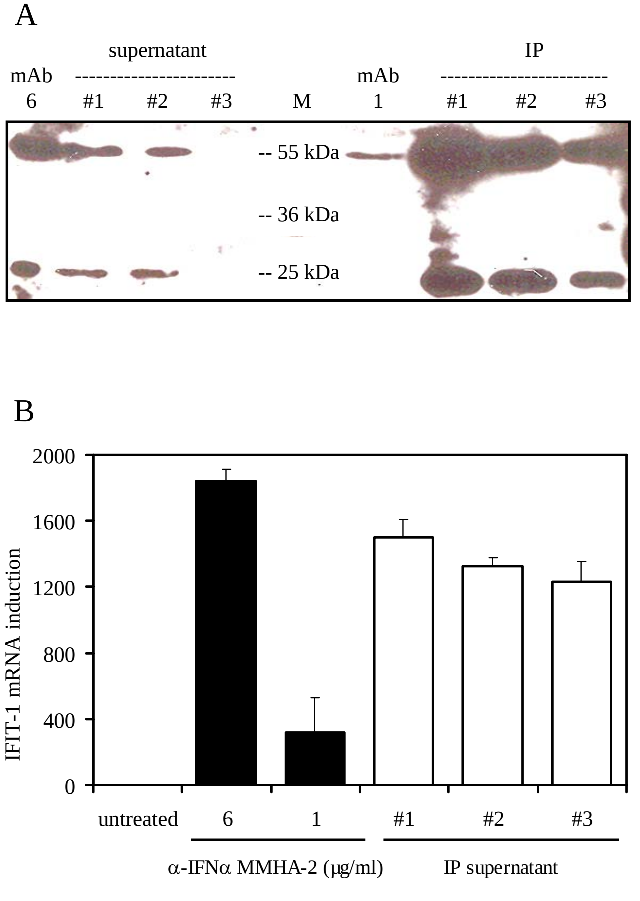

Supplementary figure 2

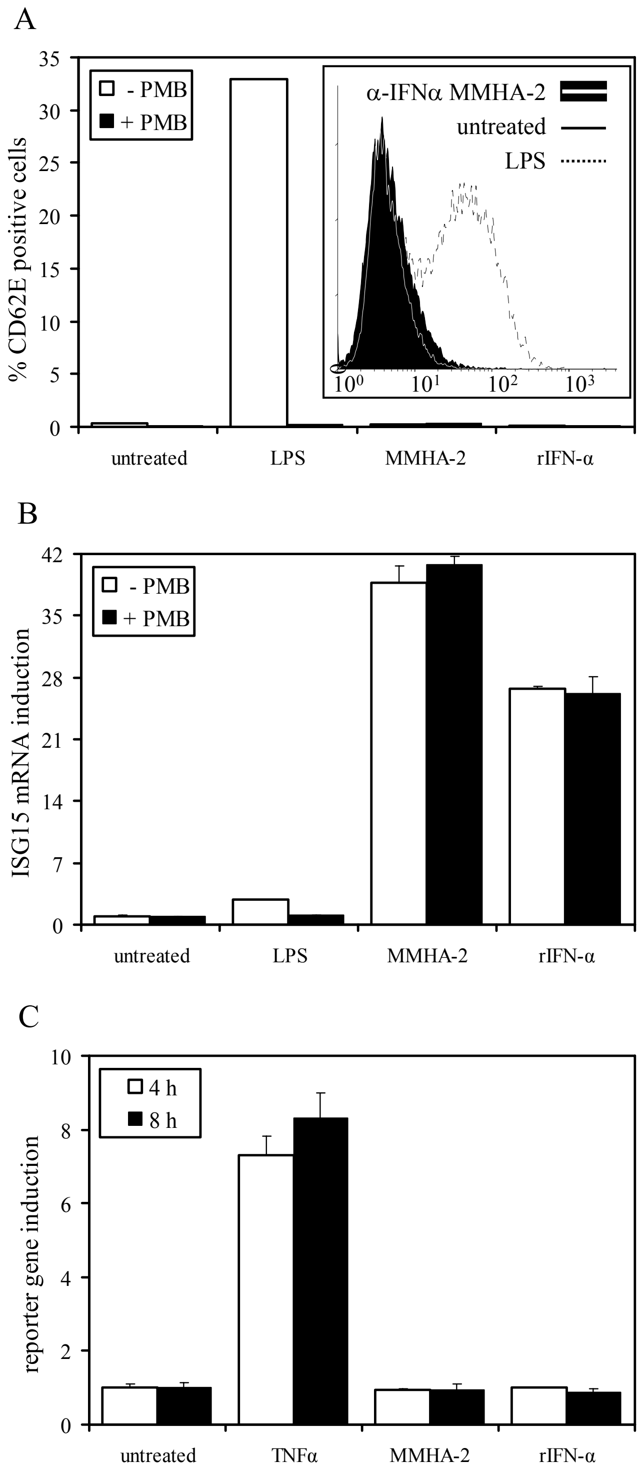

Supplementary figure 3

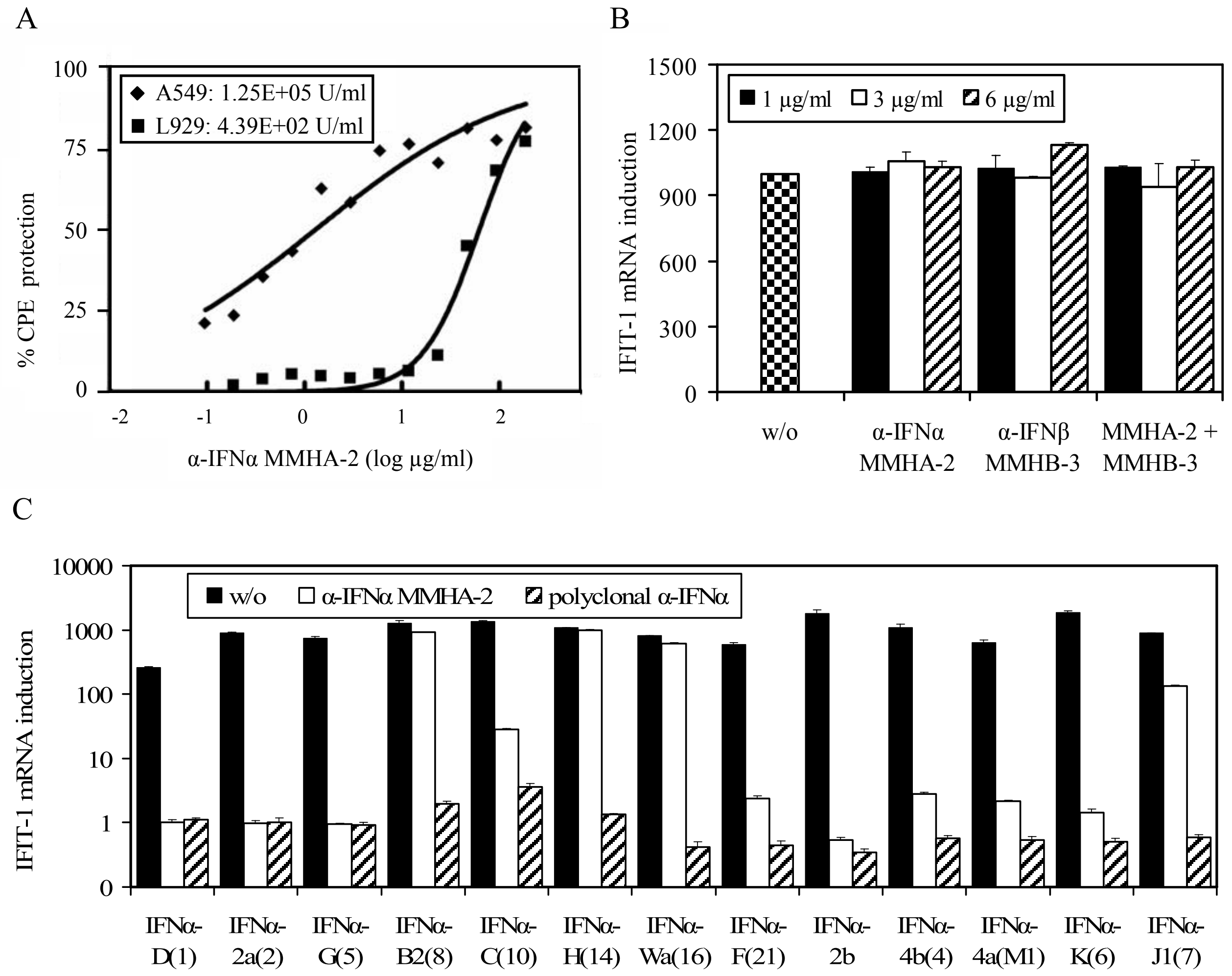

Supplementary Table 1. Detection of rIFN-α subtypes by ELISA[a)]

Sample	Input (pg/ml)	ELISA #41100 human IFN-α (pg/ml)	Detection (%)	ELISA #41105 human IFN-α (pg/ml)	Detection (%)
rIFN-α_{2a}	10 000	10 000	100.0	9 887	99
rIFN-α_{8}	10 000	0	0.0	1 035	10
rIFN-α_{14}	10 000	20	0.2	385	4
α-IFN-α MMHA-2	unknown	730		3 083	

[a)] The sensitivity of commercially available ELISA variants (PBL #41100 and #41105) towards different subtypes of rIFN-α was evaluated in comparison to the unknown contaminant in the preparation of α-IFN-α mAb MMHA-2. To avoid interference by the antibody, the contaminant was separated from the monoclonal by protein G column. The flow-through was applied to the ELISA tests and represents a 10-fold dilution of the starting material.
